# Risk factors to identify the indication for regional nodal irradiation in T1-2N1M0 breast cancer: A joint analysis of 4,243 real-world cases from two institutions

**DOI:** 10.3389/fonc.2022.955381

**Published:** 2022-12-20

**Authors:** Guang-Yi Sun, Ge Wen, Yu-Jing Zhang, Yu Tang, Hao Jing, Hui Fang, Jian-Yang Wang, Jiang-Hu Zhang, Xu-Ran Zhao, Si-Ye Chen, Yong-Wen Song, Jing Jin, Yue-Ping Liu, Yuan Tang, Shu-Nan Qi, Ning Li, Bo Chen, Ning-Ning Lu, Ye-Xiong Li, Shu-Lian Wang

**Affiliations:** ^1^ Department of Radiation Oncology, National Cancer Center/National Clinical Research Center for Cancer/Cancer Hospital, Chinese Academy of Medical Sciences and Peking Union Medical College, Beijing, China; ^2^ Department of Radiation Oncology, Sun Yat-sen University Cancer Center, State Key Laboratory of Oncology in South China, Collaborative Innovation Center of Cancer Medicine, Guangzhou, Guangdong, China; ^3^ Department of Radiation Oncology, The Third Affiliated Hospital of Guangzhou Medical University, Guangzhou, China

**Keywords:** breast neoplasm, regional nodal irradiation, one to three positive nodes, radiotherapy, risk factor

## Abstract

**Purpose:**

The aim of this study is to evaluate the role of regional nodal irradiation (RNI) in patients with T1-2N1M0 breast cancer and to identify the subgroup that could benefit from RNI.

**Methods and materials:**

A total of 4,243 women with pT1-2N1M0 breast cancer treated at two institutions in China were retrospectively reviewed. Survival rates were calculated by the Kaplan–Meier method and compared by the log-rank test. The association of risk factors with survival outcomes was evaluated using multivariable proportional hazards regression.

**Results:**

A total of 932 patients (22.0%) received RNI. At a median follow-up of 5.9 years, the 5-year locoregional recurrence (LRR), distant metastasis (DM), disease-free survival (DFS), and overall survival (OS) rates were 4.0% and 7.2% (*P* = 0.001), 13.2% and 10.6% (*P* = 0.465), 85.0% and 84.7% (*P* = 0.131), and 93.9% and 92.8% (*P* = 0.004) in the RNI and non-RNI groups, respectively. Multivariate analysis revealed that RNI was an independent prognostic factor for lower LRR (*P* = 0.001) and longer DFS (*P* = 0.013). Patients were stratified into low-, intermediate-, and high-risk groups based on the eight non-therapeutic risk factors. RNI significantly decreased the 5-year LRR (2.2% vs. 7.0%, *P* = 0.001) and improved the 5-year DFS (88.8% vs. 84.9%, *P* = 0.015) and OS (95.8% vs. 93.9%, *P* = 0.010) in the intermediate-risk group. However, neither the low-risk group nor the high-risk group had survival benefit from RNI.

**Conclusion:**

T1-2N1M0 breast cancer is a heterogeneous disease. We found that RNI only improved survival in the intermediate-risk group. It might be omitted in low-risk patients, and the role of RNI in high-risk patients needs further study.

## Introduction

Postmastectomy radiotherapy (PMRT), which commonly includes chest wall and regional nodal irradiation (RNI), has been recommended in patients with node-positive breast cancer after mastectomy, whereas whole-breast irradiation (WBI) has been generally recommended in early-stage patients after breast-conserving surgery (BCS). Both PMRT and WBI have been confirmed to reduce the risk of locoregional recurrence (LRR) and improve breast cancer-specific survival (1, 2).

In the context of modern surgical techniques and enhanced systemic therapy, the role of PMRT or RNI in addition to WBI remains controversial in patients with T1-2N1M0 breast cancer who have undergone mastectomy or BCS plus axillary lymph node dissection. The LRR rate was lower than 10% at 5 years and 11.2%–15.9% at 10 years (3–9). Some studies showed that PMRT or RNI in addition to WBI reduced LRR (6, 8), whereas other studies showed that PMRT had no impact on LRR (10–12), breast cancer-specific survival (13), or overall survival (OS) (10, 14). A study on data from the National Cancer Database (NCDB) and the Surveillance, Epidemiology, and End Results (SEER) registry showed that PMRT improved OS only in select patients, such as those with three positive nodes or two positive nodes and T2 tumors (15). However, neither database included data on tumor quadrant, biological markers, or lymphovascular invasion (LVI), which have been reported as risk factors for LRR (5, 8, 10, 16). Therefore, we retrospectively analyzed patients with T1-2N1M0 breast cancer to compare the survival outcomes between those treated with RNI and without RNI and attempted to identify the subgroup that could benefit from RNI based on the clinically available risk factors.

## Methods and materials

### Patient selection

Patients with pT1-2N1M0 breast cancer treated at two institutions in China between January 1999 and December 2014 were recruited for the study. The study protocol was approved by the Institutional Review Board of Cancer Hospital, Chinese Academy of Medical Sciences and Peking Union Medical College (approval number 15-057/984). The requirement of informed consent was waived. Patients were eligible if: 1) they underwent upfront surgical management without the receipt of any prior systemic therapy; 2) all surgery consisted of either modified radical mastectomy or BCS with axillary lymph node dissection; 3) primary tumors were 5 cm or less in diameter with one to three positive axillary lymph nodes; 4) all received postoperative radiotherapy after BCS; 5) distant metastasis (DM) is absent at initial diagnosis. Physical examination and imaging examination like mammography and ultrasound were usually used for the diagnostic work-up of these patients at initial diagnosis. Clinicopathological data were recorded, including age, date of surgery, tumor morphology, histological grade, nodal status, estrogen receptor (ER) status, progesterone receptor (PR) status, human epidermal growth factor receptor 2 (HER2) status, and information from adjuvant treatments.

### Statistical analyses

After adjuvant treatments were finished, a follow-up was conducted regularly and continued until death or loss to follow-up. The general characteristics of the subjects are expressed as frequencies and percentages and compared using the Fisher exact or χ^2^ test. LRR was defined as recurrence on the ipsilateral chest wall/breast, ipsilateral axilla, supra/infraclavicular regions, and internal mammary lymph nodes (IMNs). DM was defined as clinical evidence of distant disease based on clinical and/or radiographic findings. Disease-free survival (DFS) and OS rates were calculated from the date of the definitive surgery. Survival rates were calculated using the Kaplan–Meier method and compared by the log-rank test. The association of survival outcomes with potential prognostic factors was tested by univariate Cox regression analysis and further evaluated using multivariable proportional hazards regression. Statistical analyses were performed using the SPSS package for Windows, version 23.0 (SPSS, Inc., Chicago, IL, USA). A *P*-value of ≤0.05 was considered statistically significant.

## Results

### Patient characteristics

**Figure 1:**
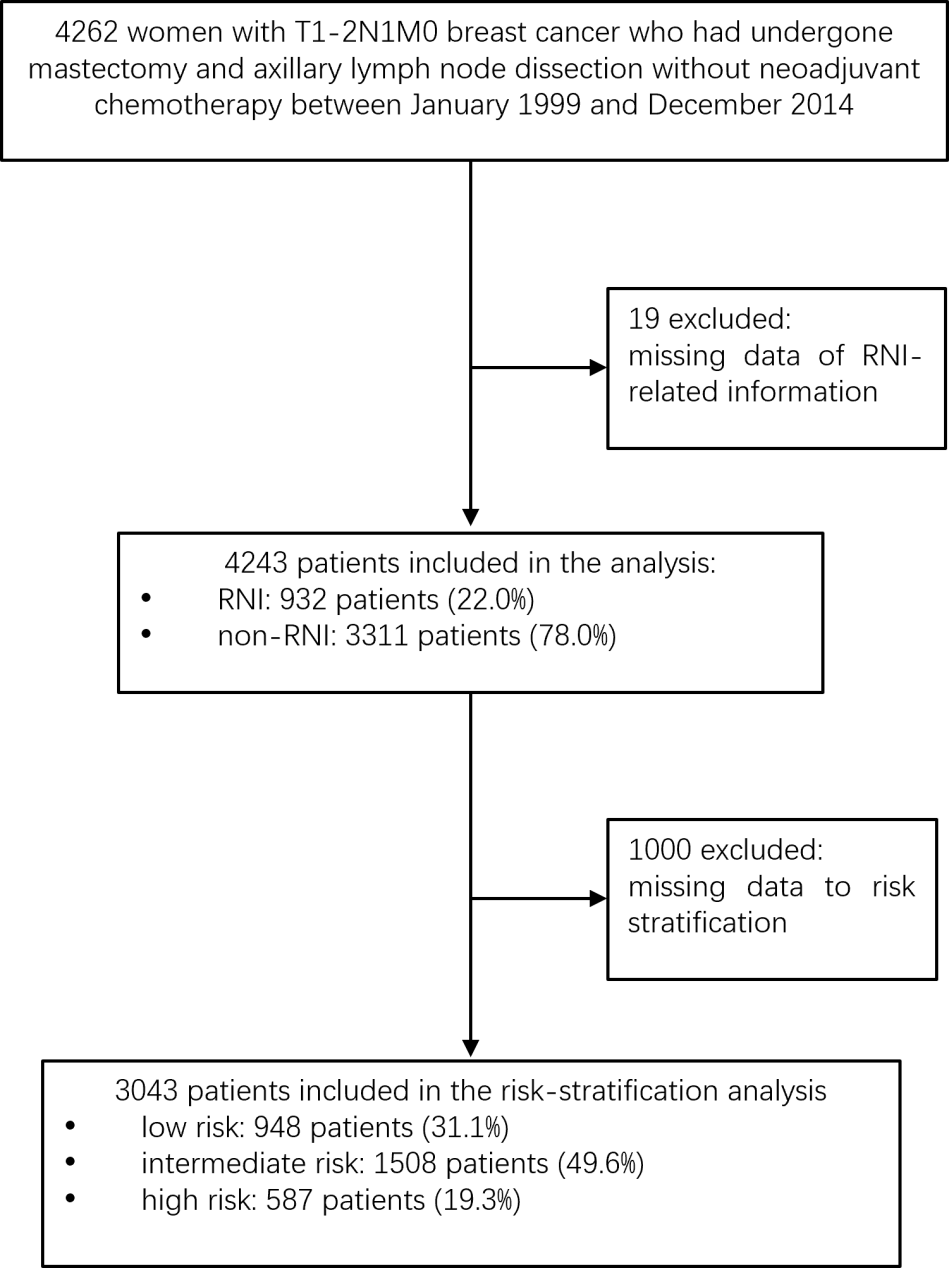
Flow chart.

After excluding 19 patients for whom RNI-related information was unavailable, 4,243 women were included (**Figure 1**). **Table 1** shows the demographic, tumor, and treatment characteristics of the entire patient cohort. The median age was 48 years (range, 23–84 years). A total of 3,858 (90.9%) patients received mastectomy and 385 (9.1%) received BCS. The median number of axillary lymph nodes removed was 17 [interquartile range (IQR), 13–22]. The median number of positive nodes was 1 (range, 1–3). A total of 3,976 out of the 4,243 (93.7%) patients received adjuvant chemotherapy with a median of six cycles (range, 1–16). Of the 3,279 out of the 4,243 (77.3%) patients with ER-/PR-positive tumors, 2,845 (86.8%) received hormonal therapy. Of the 857 out of the 4,243 (20.2%) patients with HER2-positive tumors, 231 (27.0%) received adjuvant trastuzumab.

**Table 1:** Baseline characteristics of the entire patient cohort.

	No. (%)			*P*
	Entire cohort (n = 4,243)	Non-RNI (n = 3,311)	RNI (n = 932)	
Year				< 0.001
1999–2008	1,975 (46.5)	1,613 (48.7)	362 (38.8)	
2009–2014	2,268 (53.5)	1,698 (51.3)	570 (61.2)	
Age (years)				<0.001
≤40	796 (18.8)	545 (16.5)	251 (26.9)	
>40	3,447 (81.2)	2,766 (83.5)	681 (73.1)	
Tumor location				0.777
Inner quadrant	906 (21.4)	714 (21.6)	192 (20.6)	
Other quadrants	3,277 (77.2)	2,551 (77.0)	726 (77.9)	
Unknown	60 (1.4)	46 (1.4)	14 (1.5)	
T stage				< 0.001
T1	1,996 (47.0)	1,605 (48.5)	391 (42.0)	
T2	2,247 (53.0)	1,706 (51.5)	541 (58.0)	
No. of positive nodes				<0.001
1	2,186 (51.5)	1,877 (56.7)	309 (33.2)	
2–3	2,057 (485)	1,434 (43.3)	623 (66.8)	
Lymphovascular invasion				<0.001
No	3,439 (81.1)	2,717 (82.1)	722 (77.5)	
Yes	521 (12.3)	357 (10.8)	164 (17.6)	
Unknown	283 (6.6)	237 (7.2)	46 (4.9)	
Histological grade				<0.001
I	131 (3.1)	117 (3.5)	14 (1.5)	
II	2,278 (53.7)	1,809 (54.6)	469 (50.3)	
III	1,026 (24.2)	784 (23.7)	242 (26.0)	
Unknown	808 (19.0)	601 (18.2)	207 (22.2)	
Hormonal receptor				<0.001
Negative	911 (21.5)	667 (20.1)	244 (26.2)	
Positive	3279 (77.3)	2610 (78.8)	669 (71.8)	
Unknown	53 (1.2)	34 (1.0)	19 (2.0)	
HER2				<0.001
Negative	2,830 (66.7)	2,259 (68.2)	571 (61.3)	
Positive	857 (20.2)	641 (19.4)	216 (23.2)	
Unknown	556 (13.1)	411 (12.4)	145 (15.6)	
Surgery				0.002
Breast-conserving	385 (9.1)	276 (8.3)	109 (11.7)	
Mastectomy	3,858 (90.9)	3,035 (91.7)	823 (88.3)	
Chemotherapy				< 0.001
No	239 (5.6)	229 (6.9)	10 (1.1)	
Yes	3,976 (93.7)	3,056 (92.3)	920 (98.7)	
Unknown	28 (0.7)	26 (0.8)	2 (0.2)	
Hormonal therapy				0.110
No	1,107 (26.1)	846 (25.6)	261 (28.0)	
Yes	3,004 (70.8)	2,368 (71.5)	636 (68.2)	
Unknown	132 (3.1)	97 (2.9)	35 (3.8)	
Target therapy				<0.001
No	3,438 (81.0)	2,744 (82.9)	694 (74.5)	
Yes	231 (5.4)	142 (4.3)	89 (9.5)	
Unknown	574 (13.5)	425 (12.8)	149 (16.0)	

RNI, regional nodal irradiation; HER2, human epidermal growth factor receptor 2.

Among 3,858 patients who underwent mastectomy, 832 (21.6%) received PMRT, with 823 (98.9%) receiving chest wall irradiation plus RNI and 9 (1.1%) receiving chest wall irradiation only. All 385 patients who underwent BCS received postoperative radiotherapy. Of them, 109 (28.3%) received WBI plus RNI, and 276 (71.7%) received WBI only. In the 932 patients who received RNI, the supra-/infraclavicular nodal region, axilla, and IMNs were irradiated in 928 (99.6%), 52 (5.6%), and 82 (8.8%) patients, respectively. In the 1,217 patients who received radiotherapy, the total dose was 50 Gy in 25 fractions or 48.6 Gy in 27 fractions in 1,130 (92.9%) patients or 40 Gy in 15 fractions or 43.5 Gy in 15 fractions in 87 (7.1%) patients. An additional tumor bed boost of 8.7 Gy in three fractions or 10 Gy in five fractions was delivered after WBI in 366 out of the 385 (95.1%) patients who underwent BCS. Information on radiation techniques was available for a total of 751 out of the 1,217 (61.7%) patients, of which 568 (75.6%), 27 (3.6%), and 156 (20.8%) received two-dimensional, three-dimensional conformal, and intensity-modulated radiotherapy, respectively.

Patients were classified into two groups: the RNI group (n = 932, 22.0%) and the non-RNI group (n = 3,311, 78.0%). As shown in **Table 1**, compared with the non-RNI group, the RNI group had more patients treated between 2009 and 2014, aged ≤40 years, with T2 disease, two to three positive nodes, LVI, grade 3 tumors, hormonal receptor negative, and HER2-positive disease. More patients received mastectomy, chemotherapy, and anti-HER2 targeted therapy in the RNI group than that in the non-RNI group.

### The role of RNI in the entire cohort

**Figure 2:**
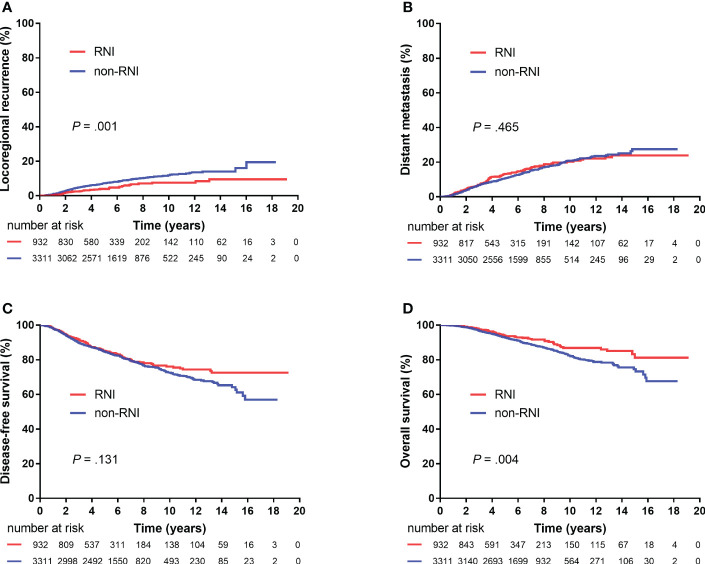
Kaplan–Meier plots showing locoregional recurrence **(A)**, distant metastasis **(B)**, disease-free survival **(C)**, and overall survival **(D)** between the RNI and non-RNI groups (4,243 patients).

The median follow-up period was 5.9 (IQR, 4.1–8.4) years. A total of 73 (1.7%), 190 (4.5%), and 68 (1.6%) patients showed local, regional, and both local and regional recurrence, respectively. Six hundred (14.1%) patients showed DMs, and 442 (10.4%) patients died. The 5-year LRR, DM, DFS, and OS rates for the entire cohort were 6.6%, 11.1%, 84.8%, and 93.1%, respectively. Furthermore, the 5-year LRR, DM, DFS, and OS rates were 4.0% and 7.2% (*P* = 0.001), 13.2% and 10.6% (*P* = 0.465), 85.0% and 84.7% (*P* = 0.131), and 93.9% and 92.8% (*P* = 0.004) in the RNI and non-RNI groups, respectively (**Figure 2**).

The results of the univariate and multivariate analyses of risk factors for survival outcomes are shown in **Table 2**. RNI was an independent risk factor for lower LRR [hazard ratio (HR), 0.41; 95% confidence interval (CI), 0.26–0.63; *P* < 0.00] and higher DFS (HR, 0.73; 95% CI, 0.57–0.94; *P* = 0.013).

**Table 2:** Univariate and multivariate analyses of risk factors for outcome events.

Variate	Locoregional recurrence				Distant metastasis				Disease-free survival				Overall survival			
	UnivariateHR (95% CI)	*P*	MultivariateHR (95% CI)	*P*	UnivariateHR (95% CI)	*P*	MultivariateHR (95% CI)	*P*	UnivariateHR (95% CI)	*P*	MultivariateHR (95% CI)	*P*	UnivariateHR (95% CI)	*P*	MultivariateHR (95% CI)	*P*
Year																
1999–2008	1.00		1.00		1.00		1.00		1.00		1.00		1.00		1.00	
2009–2014	0.77 (0.61–0.98)	0.034	1.13 (0.84–1.52)	0.416	0.79 (0.66–0.94)	0.008	0.94 (0.75–1.17)	0.579	0.73 (0.62–0.85)	<0.001	0.88 (0.73–1.07)	0.191	0.66 (0.53–0.83)	<0.001	0.78 (0.59–1.03)	0.079
Age (years)																
>40	1.00		1.00		1.00		1.00		1.00		1.00		1.00		1.00	
≤40	1.48 (1.16–1.90)	0.002	1.53 (1.11–2.12)	0.009	1.28 (1.06–1.55)	0.011	1.47 (1.16–1.86)	0.002	1.24 (1.05–1.47)	0.011	1.41 (1.14–1.75)	0.001	1.17 (0.93–1.47)	0.178	1.46 (1.08–1.97)	0.013
Tumor location																
Others	1.00		1.00		1.00		1.00		1.00		1.00		1.00		1.00	
Inner quadrant	2.02 (1.60–2.54)	<0.001	1.92 (1.44–2.56)	<0.001	1.53 (1.28–1.83)	<0.001	1.47 (1.18–1.84)	0.001	1.49 (1.28–1.74)	<0.001	1.46 (1.20–1.77)	<0.001	1.40 (1.14–1.73)	0.002	1.29 (0.98–1.71)	0.069
T stage																
T1	1.00		1.00		1.00		1.00		1.00		1.00		1.00		1.00	
T2	2.24 (1.77–2.84)	<0.001	1.95 (1.45–2.64)	<0.001	1.75 (1.48–2.08)	<0.001	1.59 (1.28–1.96)	<0.001	1.78 (1.54–2.06)	<0.001	1.59 (1.32–1.91)	<0.001	1.80 (1.47–2.19)	<0.001	1.58 (1.22–2.05)	0.001
No. of positive nodes																
1	1.00		1.00		1.00		1.00		1.00		1.00		1.00		1.00	
2–3	1.48 (1.19–1.84)	<0.001	1.85 (1.40–2.46)	<0.001	1.75 (1.48–2.08)	0.002	1.29 (1.05–1.59)	0.014	1.23 (1.07–1.41)	0.003	1.24 (1.04–1.49)	0.017	1.23 (1.02–1.49)	0.027	1.17 (0.91–1.51)	0.215
Lymphovascular invasion																
No	1.00		1.00		1.00		1.00		1.00		1.00		1.00		1.00	
Yes	1.42 (1.03–1.95)	0.033	1.37 (0.94–1.98)	0.102	1.30 (1.02–1.67)	0.037	1.38 (1.04–1.84)	0.025	1.23 (0.99–1.53)	0.064	1.25 (0.97–1.61)	0.086	1.14 (0.83–1.57)	0.414	1.09 (0.74–1.61)	0.653
Histological grade																
I– II	1.00		1.00		1.00		1.00		1.00		1.00		1.00		1.00	
III	1.63 (1.26–2.11)	<0.001	1.43 (1.08–1.90)	0.013	1.32 (1.08–1.60)	0.005	1.10 (0.89–1.37)	0.381	1.39 (1,17–1.64)	<0.001	1.13 (0.93–1.36)	0.216	1.59 (1.26–2.01)	<0.001	1.20 (0.93–1.57)	0.164
Hormone receptor																
Positive	1.00		1.00		1.00		1.00		1.00		1.00		1.00		1.00	
Negative	2.13 (1.69–2.67)	<0.001	2.01 (1.29–3.13)	0.002	1.68 (1.41–2.00)	<0.001	1.70 (1.21–2.39)	0.002	1.86 (1.60–2.16)	<0.001	1.82 (1.36–2.42)	<0.001	2.09 (1.71–2.55)	<0.001	1.82 (1.25–2.66)	0.002
HER2																
Negative	1.00		1.00		1.00		1.00		1.00		1.00		1.00		1.00	
Positive	1.67 (1.30–2.15)	<0.001	1.47 (1.06–2.03)	0.020	1.16 (0.94–1.42)	0.159	1.18 (0.92–1.53)	0.192	1.34 (1.13–1.59)	0.001	1.29 (1.04–1.60)	0.021	1.36 (1.08–1.72)	0.010	1.32 (0.98–1.78)	0.069
Surgery type																
Breast-conserving	1.00		1.00		1.00		1.00		1.00		1.00		1.00		1.00	
Mastectomy	2.36 (1.38–4.04)	0.002	1.58 (0.89–2.81)	0.117	1.63 (1.61–2.28)	0.004	1.08 (0.76–1.55)	0.657	1.98 (1.44–2.71)	<0.001	0.73 (0.57–1.12)	0.242	1.91 (1.24–2.93)	0.003	1.04 (0.66–1.65)	0.864
RNI																
No	1.00		1.00		1.00		1.00		1.00		1.00		1.00		1.00	
Yes	0.60 (0.43–0.82)	0.001	0.41 (0.26–0.63)	<0.001	1.08 (0.88–1.31)	0.465	0.83 (0.63–1.09)	0.186	0.87 (0.73–1.04)	0.131	0.73 (0.57–0.94)	0.013	0.68 (0.52–0.89)	0.004	0.74 (0.51–1.06)	0.102
Chemotherapy																
No	1.00		1.00		1.00		1.00		1.00		1.00		1.00		1.00	
Yes	1.31 (0.77–2.24)	0.320	1.74 (0.77–3.93)	0.185	0.90 (0.65–1.27)	0.561	0.92 (0.58–1.48)	0.744	0.66 (0.51–0.85)	0.001	0.70 (0.49–1.00)	0.053	0.42 (0.31–0.56)	<0.001	0.39 (0.26–0.58)	<0.001
Hormonal therapy																
No	1.00		1.00		1.00		1.00		1.00		1.00		1.00		1.00	
Yes	0.66 (0.52–0.83)	<0.001	1.05 (0.69–1.61)	0.810	0.67 (0.56–0.80)	<0.001	0.89 (0.65–1.23)	0.484	0.63 (0.54–0.73)	<0.001	0.84 (0.64–1.10)	0.836	0.49 (0.41–0.60)	<0.001	0.57 (0.40–0.82)	0.002
Target therapy																
No	1.00		1.00		1.00		1.00		1.00		1.00		1.00		1.00	
Yes	0.76 (0.42–1.35)	0.341	0.54 (0.28–1.05)	0.069	0.63 (0.39–1.01)	0.053	0.53 (0.31–0.91)	0.022	0.57 (0.38–0.88)	0.010	0.48 (0.30–0.78)	0.003	0.38 (0.18–0.81)	0.009	0.31 (1.13–0.73)	0.007

HR, hazard ratio; CI, confidence interval; RNI, regional nodal irradiation; HER2, human epidermal growth factor receptor 2.

### The role of RNI in different risk groups

**Figure 3:**
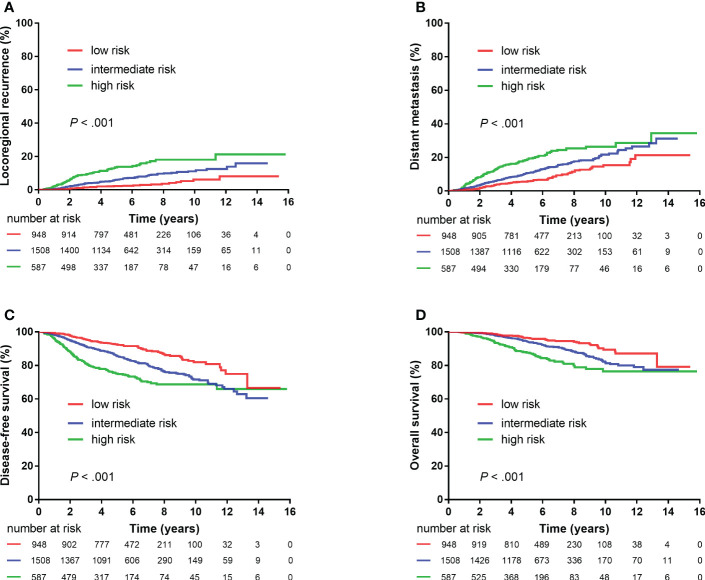
Kaplan–Meier plots showing locoregional recurrence **(A)**, distant metastasis **(B)**, disease-free survival **(C)**, and overall survival **(D)** among different risk groups (3,043 patients).

The following eight non-therapeutic risk factors proven to independently predict either LRR, DM, DFS, or OS in multivariate analysis were used to define risk groups: age (≤40 years vs.>40 years), tumor location (inner quadrant vs. other quadrants), pT stage (T2 vs. T1), number of positive nodes (two to three vs. one), LVI (yes vs. no), histological grade (grade 3 vs. grades 1–2), hormonal receptor (negative vs. positive), and HER2 status (positive vs. negative). Out of the 4,243 patients, 3,043 (71.7%) patients who had information available on these factors were stratified into three risk groups: low-risk [0–1 risk factors, n = 948 (31.1%)], intermediate-risk [2–3 risk factors, n = 1,508 (49.6%)], and high-risk [≥4 risk factors, n = 587 (19.3%)] groups. Significant differences were observed in LRR, DM, DFS, and OS among the three risk groups (all *P* < 0.001, **Figure 3**). After adjusting for treatment, the risk group was independently associated with LRR, DM, DFS, and OS in multivariate analysis (**Table 3**).

**Table 3:** Multivariate analyses of risk group adjusted by era and treatments.

Variate	Locoregional recurrence		Distant metastasis		Disease-free survival		Overall survival	
	HR (95% CI)	*P*	HR (95% CI)	*P*	HR (95% CI)	*P*	HR (95% CI)	*P*
Risk group								
Low-risk	1.00		1.00		1.00		1.00	
Intermediate-risk	2.46 (1.63–3.70)	<0.001	1.61 (1.24–2.09)	<0.001	1.76 (1.39–2.22)	<0.001	1.60 (1.15–2.22)	0.005
High-risk	5.42 (3.46–8.49)	<0.001	2.60 (1.91–3.55)	<0.001	2.70 (2.05–3.56)	<0.001	2.50 (1.71–3.66)	<0.001
Year								
1999–2008	1.00		1.00		1.00		1.00	
2009–2014	1.09 (0.81–1.46)	0.552	0.93 (0.75–1.16)	0.545	0.88 (0.73–1.06)	0.175	0.78 (0.59–1.02)	0.074
Surgery type								
Breast-conserving	1.00		1.00		1.00		1.00	
Mastectomy	1.71 (0.97–3.02)	0.062	1.14 (0.80–1.62)	0.474	1.29 (0.93–1.80)	0.130	1.10 (0.70–1.73)	0.671
RNI								
No	1.00		1.00		1.00		1.00	
Yes	0.47 (0.30–0.71)	<0.001	0.91 (0.70–1.19)	0.491	0.79 (0.62–0.99)	0.045	0.80 (0.56–1.15)	0.235
Chemotherapy								
No	1.00		1.00		1.00		1.00	
Yes	1.88 (0.83–4.25)	0.129	0.99 (0.62–1.58)	0.969	0.75 (0.53–1.07)	0.118	0.43 (0.28–0.64)	<0.001
Hormonal therapy								
No	1.00		1.00		1.00		1.00	
Yes	0.85 (0.63–1.14)	0.273	0.73 (0.59–0.92)	0.007	0.65 (0.54–0.79)	<0.001	0.43 (0.33–0.56)	<0.001
Target therapy								
No	1.00		1.00		1.00		1.00	
Yes	0.58 (0.31–1.08)	0.085	0.53 (0.32–0.88)	0.014	0.52 (0.33–0.81)	0.005	0.34 (0.15–0.78)	0.011

HR, hazard ratio; CI, confidence interval.

**Figure 4:**
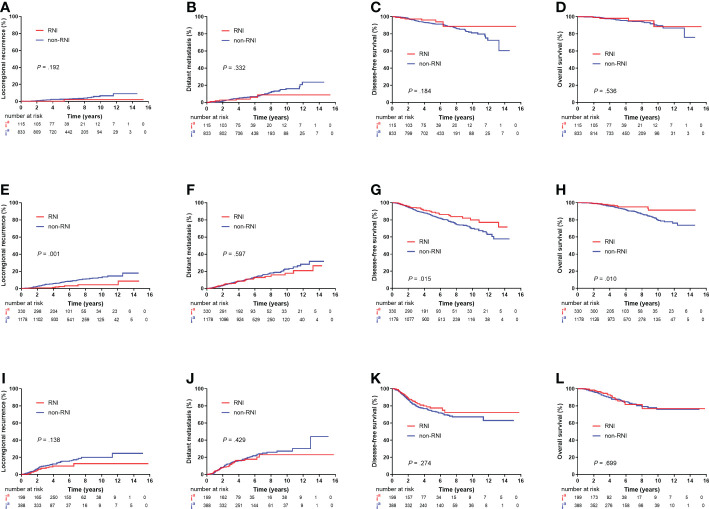
Kaplan–Meier plots showing locoregional recurrence, distant metastasis, disease-free survival, and overall survival between the RNI and non-RNI groups in low-risk patients **(A–D)**, intermediate-risk patients **(E–H)**, and high-risk patients **(I–L)**.

A total of 115 out of the 948 (12.1%) patients in the low-risk group, 220 out of the 1,508 (21.9%) patients in the intermediate-risk group, and 199 out of the 587 (33.9%) patients in the high-risk group received RNI. RNI significantly reduced 5-year LRR (2.2% vs. 7.0%, *P* = 0.001) and improved DFS (88.8% v. 84.9%, *P* = 0.015) and OS (95.8% vs. 93.9%, *P* = 0.010) in the intermediate-risk group but had no impact on survival outcomes in the low- or high-risk groups (**Figure 4**).

After adjusting for the eight non-therapeutic risk factors, treatment era, surgery type, chemotherapy, hormonal therapy, and targeted therapy, RNI was associated with lower LRR (HR, 0.22; 95% CI, 0.10–0.49; *P* < 0.001) and higher DFS (HR, 0.66; 95% CI, 0.46–0.94; *P* = 0.023) and OS (HR, 0.55; 95% CI, 0.31–0.99; *P* = 0.048) in the intermediate-risk group (**Supplementary Table 1**). In the high-risk group, RNI was only associated with lower LRR (HR, 0.56; 95% CI, 0.31–1.00; 14.9% vs. 10.8% at 5 years; *P* = 0.050) (**Supplementary Table 2**). However, the low-risk group did not benefit from RNI (**Supplementary Table 3**).

## Discussion

The present study showed that T1-2N1M0 breast cancer is a heterogeneous disease, and patients can be stratified into three groups on the basis of eight clinically available risk factors. RNI significantly decreased LRR and improved DFS and OS in the intermediate-risk group. The DFS and OS advantage from RNI among patients in the intermediate-risk group was ~4% and ~2% at 5 years. However, RNI only decreased LRR in the high-risk group in multivariate analysis and had no effect on the low-risk group. The eight non-therapeutic risk factors may, therefore, be useful as predictors for potential OS benefit from RNI in patients with T1-2N1M0 breast cancer.

The value of RNI in patients with T1-2N1M0 breast cancer remains a topic of great clinical relevance and controversy. Our results showed that the 5-year LRR was 7.2% in patients with T1-2N1M0 breast cancer without RNI, which is consistent with other contemporary studies showing that the 5-year LRR was less than 10% (9, 17–19). Given the low LRR risk, the necessity of RNI in T1-2N1M0 breast cancer has been questioned. The results of the EORTC 22922 and MA.20 randomized studies suggested that RNI improved DFS independent of its effect on LRR, and the absolute 10-year DFS benefit from RNI was 3% and 5% (20, 21). In these two studies, RNI decreased DM significantly, indicating that RNI may eradicate locoregional areas of disease not destroyed by systemic therapy and that these areas could be the sources of eventual tumor dissemination, although active disease may not clinically manifest at those locoregional sites before or after systemic relapse. The benefit of RNI should be evaluated by looking beyond LRR rates. Therefore, we used the eight non-therapeutic risk factors identified for either LRR, DM, DFS, or OS to stratify patients into three risk groups with different prognoses, which is consistent with the findings of another study showing that the concomitant presence of multiple risk factors contributes to higher tumor recurrence (9).

In the low-risk group, the risk of recurrence was very low, and there was no benefit from RNI in terms of any endpoints; therefore, RNI may be omitted. Avoiding unnecessary RNI might reduce the risk of toxicities such as hypothyroidism (22), pneumonitis (20), lymphedema (20), or cardiac disease (23) that were resulted from RNI. Similarly, Bazan et al. and Muhsen et al. found that most patients with T1-2N1M0 breast cancer with low tumor burdens had low event rates, such that PMRT could have been omitted (24, 25). In contrast, an observational cohort study was performed on patients with T1-2N1 ER-positive breast cancer from the NCDB and the SEER registry who underwent mastectomy, and the result showed that the longer survival associated with radiotherapy was limited to patients with a low Oncotype Dx score, which indicates the complicated competitive relationship between local and distant relapse and suggests that the risk group differentiated solely by clinical factors may be insufficient (26). The ongoing MA 39/TAILOR RT trial selected HR+HER2-negative population with low Oncotype Dx score and randomized them to receive PMRT or not (NCT03488693). The result of the study is expected to answer the question of the role of RNI in the low-risk group.

In the present study, the effect of RNI on LRR and the translation from LRR benefit to survival benefit differed between the intermediate-risk and high-risk groups. The relative reduction of LRR from RNI was greater in the intermediate-risk group than that in the high-risk group (HR 0.22 vs. 0.56). The intermediate-risk group showed survival benefits from RNI in multivariate analysis, thus select use of RNI in the intermediate-risk group is recommended. Whereas RNI had no impact on survival in the high-risk group, significant decrease in LRR was achieved. A previous meta-analysis showed that comprehensive chest wall and RNI reduced LRR by two-thirds and that the survival benefit was proportional to the absolute reduction of LRR^2^. Our findings showing that RNI was less effective in the high-risk group might first be explained by the competing risk of distant disease recurrence in the high-risk group that might negate survival benefits from RNI. The systemic therapy was insufficient, e.g., anti-HER2 targeted therapy was administered in only 27% of patients with HER2-positive disease. Second, the lower use of INM irradiation might reduce the efficacy of RNI. Given the potential increased dose to the heart and lung and unconfirmed OS benefit from additional IMN irradiation in randomized trials (20, 27, 28), we did not routinely irradiate IMN in prior clinical practice. However, we found that medial tumors were independently associated with poorer survival. A few studies have consistently shown that medial breast cancers carry a worse prognosis than lateral breast cancers, even after adjusting for other known prognostic factors (5, 29, 30). Because IMN involvement occurred more frequently in patients with medially located tumors (31), coverage of IMN in RNI might be considered. One previous study showed that supraclavicular or IMN irradiation alone did not improve survival outcomes in N1 breast cancer (18). A Danish study showed that additional IMN irradiation significantly improved the OS in patients with one to three positive nodes and medially located tumors (32). A meta-analysis of four prospective trials revealed that supra-/infraclavicular nodal irradiation reduced LRR without improvement in OS, and a prolongation in survival due to RNI is achieved when the IMN is included, based on a reduction in DM (33). Therefore, we assumed that the high-risk group might expect benefit from RNI if systemic therapy is sufficient and IMN is included. In contrast, Luo et al. reported that patients with T1-2N1 breast cancer were sorted into three risk groups with distinct LRRs on the bases of five risk factors, namely, T2 disease, three positive nodes, grade 3 disease, ER-negative status, and presence of LVI. PMRT was significantly correlated with decreased LRR, DM, and breast cancer mortality in the high-risk group (34). Wu et al. restaged T1-2N1 breast cancer from the SEER registry on the basis of the eighth edition of the American Joint Committee on Cancer (AJCC) pathological staging system and found that PMRT was only associated with better BCSS in the high-risk group, i.e., stage IIIA disease (35). However, details regarding the radiation fields used for the patients analyzed were not provided in these two studies.

To our knowledge, the present study is one of the few studies to investigate the role of RNI in patients with T1-2N1M0 stage breast cancer. An important strength of our study was the inclusion of data from two large cancer centers to thoroughly investigate the role of RNI by risk stratification. Furthermore, our study accounts for as many confounding factors as possible.

This study was designed with reference to most randomized or prospective studies like EORTC 22922, KROG 08-06, and Danish study, in which RNI was evaluation in patients who had undergone BCS and mastectomy (21, 32, 36). However, the study is subject to the same limitations of a retrospective study. First, patients with high-risk factors were chosen to receive RNI, which only accounted for 22% of the entire cohort. Adjustment for registered characteristics might not exclude residual confounding, and selection bias still potentially influenced the estimation of treatment effects. Second, with advancements in diagnostic and therapeutic strategies, treatment guidelines for patients with breast cancer have changed over the time period that this study covers, and the findings may not fully reflect the outcomes of patients currently being treated. Specifically, this cohort appeared to receive more intensive surgery and chemotherapy but less endocrine therapy and anti-HER2 targeted therapy than would be expected in the modern era. For example, over 90% of patients in this cohort underwent mastectomy; we only included patients who received axillary lymph node dissection, rather than sentinel node biopsy; and 93.7% of patients received chemotherapy; when indicated, only 86.8% and 29% of patients received endocrine therapy and anti-HER2 targeted therapy. However, the study from NCDB registry demonstrated marked trends toward higher proportions of BCS-eligible patients undergoing mastectomy, breast reconstruction, and bilateral mastectomy (37). Thus, the findings of the present study are clinically relevant. Third, to eliminate the influence of confounding, all patients in the study had not received neoadjuvant chemotherapy that is widely used to treat stage II to III breast cancers in modern era. Further study is needed to analyze the value of RNI based on the neoadjuvant chemotherapy. Fourth, the sample sizes of the risk stratified groups become quite small and conclusions from this cohort might be tempered on the basis of the small number of patients in each group. At last, because of the limited follow-up time, the effect of RNI on survival warrants further validation.

## Conclusions

T1-2N1M0 breast cancer is a heterogeneous disease. We found that the RNI only improved survival in the intermediate-risk group. RNI might be omitted in the low-risk group, and the role of RNI in the high-risk group needs further study.

## Data availability statement

The original contributions presented in the study are included in the article/**Supplementary Material**. Further inquiries can be directed to the corresponding authors.

## Ethics statement

The studies involving human participants were reviewed and approved by Institutional Review Board of Cancer Hospital, Chinese Academy of Medical Sciences and Peking Union Medical College. The ethics committee waived the requirement of written informed consent for participation.

## Author contributions

G-YS, GW and Y-JZ: Formal analysis, investigation, data collection, methodology, and writing of the original draft. YuanT, HJ, HF, J-YW, J-HZ, Y-WS, JJ, Y-PL, YuanT, S-NQ, NL, BC and N-NL: Patient care and review and editing of the manuscript. X-RZ and S-YC: Data collection, statistics, and review and editing of the original draft. Y-XL and S-LW: Formal analysis and data collection, validation, statistical analysis guidance, project administration, patient care, and writing and editing of the first draft of the manuscript. All authors contributed to the article and approved the submitted version.

## References

[1] Darby S McGale P Correa C Taylor C Arriagada R Clarke M. Effect of radiotherapy after breast-conserving surgery on 10-year recurrence and 15-year breast cancer death: meta-analysis of individual patient data for 10,801 women in 17 randomised trials. Lancet (2011) 378(9804):1707–16. doi: 10.1016/s0140-6736(11)61629-2 PMC3254252 22019144

[2] McGale P Taylor C Correa C Cutter D Duane F Ewertz M. Effect of radiotherapy after mastectomy and axillary surgery on 10-year recurrence and 20-year breast cancer mortality: meta-analysis of individual patient data for 8135 women in 22 randomised trials. Lancet (2014) 383(9935):2127–35. doi: 10.1016/s0140-6736(14)60488-8 PMC5015598 24656685

[3] Recht A Pierce SM Abner A Vicini F Osteen RT Love SM. Regional nodal failure after conservative surgery and radiotherapy for early-stage breast carcinoma. J Clin Oncol (1991) 9(6):988–96. doi: 10.1200/JCO.1991.9.6.988 2033433

[4] Taghian A Jeong JH Mamounas E Anderson S Bryant J Deutsch M. Patterns of locoregional failure in patients with operable breast cancer treated by mastectomy and adjuvant chemotherapy with or without tamoxifen and without radiotherapy: results from five national surgical adjuvant breast and bowel project randomized clinical trials. J Clin Oncol (2004) 22(21):4247–54. doi: 10.1200/JCO.2004.01.042 15452182

[5] Truong PT Olivotto IA Kader HA Panades M Speers CH Berthelet E. Selecting breast cancer patients with T1-T2 tumors and one to three positive axillary nodes at high postmastectomy locoregional recurrence risk for adjuvant radiotherapy. Int J Radiat Oncol Biol Phys (2005) 61(5):1337–47. doi: 10.1016/j.ijrobp.2004.08.009 15817335

[6] Truong PT Jones SO Kader HA Wai ES Speers CH Alexander AS. Patients with t1 to t2 breast cancer with one to three positive nodes have higher local and regional recurrence risks compared with node-negative patients after breast-conserving surgery and whole-breast radiotherapy. Int J Radiat Oncol Biol Phys (2009) 73(2):357–64. doi: 10.1016/j.ijrobp.2008.04.034 18676091

[7] Aristei C Leonardi C Stracci F Palumbo I Luini A Viale G. Risk factors for relapse after conservative treatment in T1-T2 breast cancer with one to three positive axillary nodes: results of an observational study. Ann Oncol (2011) 22(4):842–7. doi: 10.1093/annonc/mdq470 21048040

[8] Tendulkar RD Rehman S Shukla ME Reddy CA Moore H Budd GT. Impact of postmastectomy radiation on locoregional recurrence in breast cancer patients with 1-3 positive lymph nodes treated with modern systemic therapy. Int J Radiat Oncol Biol Phys (2012) 83(5):e577–581. doi: 10.1016/j.ijrobp.2012.01.076 22560546

[9] Park HJ Shin KH Kim JH Ahn SD Kim JY Park W. Incorporating risk factors to identify the indication of post-mastectomy radiotherapy in N1 breast cancer treated with optimal systemic therapy: A multicenter analysis in Korea (KROG 14-23). Cancer Res Treat (2017) 49(3):739–47. doi: 10.4143/crt.2016.405 PMC5512365 27764904

[10] Moo TA McMillan R Lee M Ahn SD Kim JY Park W. Selection criteria for postmastectomy radiotherapy in t1-t2 tumors with 1 to 3 positive lymph nodes. Ann Surg Oncol (2013) 20(10):3169–74. doi: 10.1245/s10434-013-3117-0 23975289

[11] McBride A Allen P Woodward W Kim M Kuerer HM Drinka EK. Locoregional recurrence risk for patients with T1,2 breast cancer with 1-3 positive lymph nodes treated with mastectomy and systemic treatment. Int J Radiat Oncol Biol Phys (2014) 89(2):392–8. doi: 10.1016/j.ijrobp.2014.02.013 24721590

[12] Miyashita M Tada H Suzuki A Watanabe G Hirakawa H Amari M. Minimal impact of postmastectomy radiation therapy on locoregional recurrence for breast cancer patients with 1 to 3 positive lymph nodes in the modern treatment era. Surg Oncol (2017) 26(2):163–70. doi: 10.1016/j.suronc.2017.03.003 28577722

[13] Li FY Lian CL Lei J Wang J Hua L He ZY. Real-world impact of postmastectomy radiotherapy in T1-2 breast cancer with one to three positive lymph nodes. Ann Transl Med (2020) 8(7):489. doi: 10.21037/atm.2020.03.49 32395533 PMC7210210

[14] Nordenskjold AE Fohlin H Albertsson P Arnesson LG Chamalidou C Einbeigi Z. No clear effect of postoperative radiotherapy on survival of breast cancer patients with one to three positive nodes: a population-based study. Ann Oncol (2015) 26(6):1149–54. doi: 10.1093/annonc/mdv159 25839671

[15] Huo D Hou N Jaskowiak N Winchester DJ Winchester DP Yao K. Use of postmastectomy radiotherapy and survival rates for breast cancer patients with T1-T2 and one to three positive lymph nodes. Ann Surg Oncol (2015) 22(13):4295–304. doi: 10.1245/s10434-015-4528-x 25820998

[16] Matsunuma R Oguchi M Fujikane T Matsuura M Sakai T Kimura K. Influence of lymphatic invasion on locoregional recurrence following mastectomy: indication for postmastectomy radiotherapy for breast cancer patients with one to three positive nodes. Int J Radiat Oncol Biol Phys (2012) 83(3):845–52. doi: 10.1016/j.ijrobp.2011.08.029 22138460

[17] Yoo GS Park W Yu JI Choi DH Kim YJ Shin KH. Comparison of breast conserving surgery followed by radiation therapy with mastectomy alone for pathologic N1 breast cancer patients in the era of anthracycline plus taxane-based chemotherapy: A multicenter retrospective study (KROG 1418). Cancer Res Treat (2019) 51(3):1041–51. doi: 10.4143/crt.2018.424 PMC6639204 30384580

[18] Qi WX Cao L Xu C Zhao S Chen J. Adjuvant regional nodal irradiation did not improve outcomes in T1-2N1 breast cancer after breast-conserving surgery: A propensity score matching analysis of BIG02/98 and BCIRG005 trials. Breast (2020) 49:165–70. doi: 10.1016/j.breast.2019.11.001 PMC7375686 31812892

[19] Katz A Strom EA Buchholz TA Thames HD Smith CD Jhingran A. Locoregional recurrence patterns after mastectomy and doxorubicin-based chemotherapy: implications for postoperative irradiation. J Clin Oncol (2000) 18(15):2817–27. doi: 10.1200/JCO.2000.18.15.2817 10920129

[20] Whelan TJ Olivotto IA Parulekar WR Ackerman I Chua BH Nabid A. Regional nodal irradiation in early-stage breast cancer. N Engl J Med (2015) 373(4):307–16. doi: 10.1056/NEJMoa1415340 PMC4556358 26200977

[21] Poortmans PM Collette S Kirkove C Van Limbergen E Budach V Struikmans H. Internal mammary and medial supraclavicular irradiation in breast cancer. N Engl J Med (2015) 373(4):317–27. doi: 10.1056/NEJMoa1415369 26200978

[22] Zhao XR Fang H Jing H Tang Y Song YW Liu YP. Radiation-induced hypothyroidism in patients with breast cancer after hypofractionated radiotherapy: A prospective cohort study. Int J Radiat Oncol Biol Phys (2022), S0360–3016(22)00557-0. doi: 10.1016/j.ijrobp.2022.04.052 36306978

[23] Poortmans PM Struikmans H De Brouwer P Weltens C Fortpied C Kirkove C. Side effects 15 years after lymph node irradiation in breast cancer: Randomized EORTC trial 22922/10925. J Natl Cancer Inst (2021) 113(10):1360–8. doi: 10.1093/jnci/djab113 34320651

[24] Bazan JG Majithia L Quick AM Wobb JL Terando AM Agnese DM. Heterogeneity in outcomes of pathologic T1-2N1 breast cancer after mastectomy: Looking beyond locoregional failure rates. Ann Surg Oncol (2018) 25(8):2288–95. doi: 10.1245/s10434-018-6565-8 29916008

[25] Muhsen S Moo TA Patil S Stempel M Powell S Morrow M. Most breast cancer patients with T1-2 tumors and one to three positive lymph nodes do not need postmastectomy radiotherapy. Ann Surg Oncol (2018) 25(7):1912–20. doi: 10.1245/s10434-018-6422-9 PMC5976529 29564588

[26] Goodman CR Seagle BL Kocherginsky M Donnelly ED Shahabi S Strauss JB. 21-gene recurrence score assay predicts benefit of post-mastectomy radiotherapy in T1-2 N1 breast cancer. Clin Cancer Res (2018) 24(16):3878–87. doi: 10.1158/1078-0432.CCR-17-3169 29685878

[27] Hennequin C Bossard N Servagi-Vernat S Maingon P Dubois JB Datchary J. Ten-year survival results of a randomized trial of irradiation of internal mammary nodes after mastectomy. Int J Radiat Oncol Biol Phys (2013) 86(5):860–6. doi: 10.1016/j.ijrobp.2013.03.021 23664327

[28] Poortmans PM Weltens C Fortpied C Kirkove C Peignaux-Casasnovas K Budach V. Internal mammary and medial supraclavicular lymph node chain irradiation in stage I–III breast cancer (EORTC 22922/10925): 15-year results of a randomised, phase 3 trial. Lancet Oncol (2020) 21(12):1602–10. doi: 10.1016/s1470-2045(20)30472-1 33152277

[29] Colleoni M Zahrieh D Gelber RD Holmberg SB Mattsson JE Rudenstam CM. Site of primary tumor has a prognostic role in operable breast cancer: the international breast cancer study group experience. J Clin Oncol (2005) 23(7):1390–400. doi: 10.1200/JCO.2005.06.052 15735115

[30] Kroman N Wohlfahrt J Mouridsen HT Melbye M. Influence of tumor location on breast cancer prognosis. Int J Cancer (2003) 105(4):542–5. doi: 10.1002/ijc.11116 12712447

[31] Chen RC Lin NU Golshan M Harris JR Bellon JR. Internal mammary nodes in breast cancer: diagnosis and implications for patient management – a systematic review. J Clin Oncol (2008) 26(30):4981–9. doi: 10.1200/JCO.2008.17.4862 18711171

[32] Thorsen LB Offersen BV Dano H Berg M Jensen I Pedersen AN. DBCG-IMN: A population-based cohort study on the effect of internal mammary node irradiation in early node-positive breast cancer. J Clin Oncol (2016) 34(4):314–20. doi: 10.1200/JCO.2015.63.6456 26598752

[33] Haussmann J Budach W Tamaskovics B Bolke E Corradini S Djiepmo-Njanang FJ. Which target volume should be considered when irradiating the regional nodes in breast cancer? results of a network-meta-analysis. Radiat Oncol (2019) 14(1):102. doi: 10.1186/s13014-019-1280-6 31186015 PMC6558843

[34] Luo C Zhong X Deng L Xie Y Hu K Zheng H. Nomogram predicting locoregional recurrence to assist decision-making of postmastectomy radiation therapy in patients with T1-2N1 breast cancer. Int J Radiat Oncol Biol Phys (2019) 103(4):905–12. doi: 10.1016/j.ijrobp.2018.11.005 30419307

[35] Wu SG Wang J Lian CL Lei J Hua L Lin Q. Evaluation of the 8th edition of the American joint committee on cancer's pathological staging system in prognosis assessment and treatment decision making for stage T1-2N1 breast cancer after mastectomy. Breast (2020) 51:2–10. doi: 10.1016/j.breast.2020.02.012 32172191 PMC7375569

[36] Kim YB Byun HK Kim DY Ahn SJ Lee HS Park W. Effect of elective internal mammary node irradiation on disease-free survival in women with node-positive breast cancer: A randomized phase 3 clinical trial. JAMA Oncol (2022) 8(1):96–105. doi: 10.1001/jamaoncol.2021.6036 34695841 PMC8546620

[37] Kummerow KL Du L Penson DF Shyr Y Hooks MA. Nationwide trends in mastectomy for early-stage breast cancer. JAMA Surg (2015) 150(1):9–16. doi: 10.1001/jamasurg.2014.2895 25408966

