# Supplementary material for: Risk factors to identify the indication for regional nodal irradiation in T1-2N1M0 breast cancer: A joint analysis of 4,243 real-world cases from two institutions
Source: Front Oncol. 2022 Dec 20;12:955381. doi: 10.3389/fonc.2022.955381 (PMC9807655; doi:10.3389/fonc.2022.955381)
Supplement: Supplementary file 1 [file Table_1.docx]

Supplementary Table 1. Multivariate analyses of risk factors for outcome events in the intermediate-risk group

| **Variate** | **Locoregional recurrence** | | **Distant metastasis** | | | **Disease-free survival** | | | **Overall survival** | | |
| --- | --- | --- | --- | --- | --- | --- | --- | --- | --- | --- | --- |
|  | **HR (95% CI)** | ***P*** | **HR (95% CI)** | ***P*** | **HR (95% CI)** | | ***P*** | **HR (95% CI)** | | ***P*** |  |
| Year |  |  |  |  |  | |  |  | |  |  |
| 1999-2008 | 1.00 |  | 1.00 |  | 1.00 | |  | 1.00 | |  |  |
| 2009-2014 | 0.90 (0.59-1.36) | .619 | 0.87 (0.64-1.19) | .381 | 0.78 (0.60-1.02) | | .074 | 0.70 (0.47-1.06) | | .091 |  |
| Age (years) |  |  |  |  |  | |  |  | |  |  |
| > 40 | 1.00 |  | 1.00 |  | 1.00 | |  | 1.00 | |  |  |
| ≤ 40 | 3.13 (1.69-5.80) | <.001 | 2.50 (1.63-3.84) | <.001 | 2.15 (1.47-3.16) | | <.001 | 1.56 (0.88-2.78) | | .129 |  |
| Tumor location |  |  |  |  |  | |  |  | |  |  |
| Others | 1.00 |  | 1.00 |  | 1.00 | |  | 1.00 | |  |  |
| Inner quadrant | 3.12 (1.78-5.46) | <.001 | 1.88 (1.25-2.81) | .002 | 1.81 (1.27-2.58) | | .001 | 1.20 (0.71-2.04) | | .491 |  |
| T stage |  |  |  |  |  | |  |  | |  |  |
| T1 | 1.00 |  | 1.00 |  | 1.00 | |  | 1.00 | |  |  |
| T2 | 4.22 (2.42-7.35) | <.001 | 2.22 (1.52-3.25) | <.001 | 2.25 (1.61-3.13) | | <.001 | 1.87 (1.16-2.99) | | .010 |  |
| No. of positive nodes |  |  |  |  |  | |  |  | |  |  |
| 1 | 1.00 |  | 1.00 |  | 1.00 | |  | 1.00 | |  |  |
| 2-3 | 3.98 (2.30-6.88) | <.001 | 1.83 (1.24-2.71) | .002 | 1.71 (1.22-2.39) | | .002 | 1.37 (0.85-2.20) | | .195 |  |
| Lymphovascular invasion |  |  |  |  |  | |  |  | |  |  |
| No | 1.00 |  | 1.00 |  | 1.00 | |  | 1.00 | |  |  |
| Yes | 2.81 (1.32-5.98) | .007 | 1.84 (1.08-3.14) | .025 | 1.76 (1.10-2.82) | | .019 | 1.75 (0.91-3.38) | | .095 |  |
| Histological grade |  |  |  |  |  | |  |  | |  |  |
| I- II | 1.00 |  | 1.00 |  | 1.00 | |  | 1.00 | |  |  |
| III | 2.25 (1.31-3.86) | .003 | 1.53 (1.04-2.25) | .032 | 1.60 (1.15-2.22) | | .005 | 1.55 (0.98-2.45) | | .063 |  |
| Hormone receptor |  |  |  |  |  | |  |  | |  |  |
| Positive | 1.00 |  | 1.00 |  | 1.00 | |  | 1.00 | |  |  |
| Negative | 4.08 (1.92-8.67) | <.001 | 2.95 (1.69-5.14) | <.001 | 2.90 (1.83-4.59) | | <.001 | 2.52 (1.35-4.71) | | .004 |  |
| HER2 |  |  |  |  |  | |  |  | |  |  |
| Negative | 1.00 |  | 1.00 |  | 1.00 | |  | 1.00 | |  |  |
| Positive | 3.45 (1.92-6.21) | <.001 | 1.65 (1.05-2.60) | .029 | 2.03 (1.41-2.93) | | <.001 | 1.85 (1.11-3.09) | | .019 |  |
| Surgery type |  |  |  |  |  | |  |  | |  |  |
| Breast-conserving | 1.00 |  | 1.00 |  | 1.00 | |  | 1.00 | |  |  |
| Mastectomy | 1.51 (0.60-3.81) | .383 | 1.28 (0.73-2.25) | .394 | 1.33 (0.79-2.25) | | .281 | 1.12 (0.53-2.36) | | .764 |  |
| RNI |  |  |  |  |  | |  |  | |  |  |
| No | 1.00 |  | 1.00 |  | 1.00 | |  | 1.00 | |  |  |
| Yes | 0.22 (0.10-0.49) | <.001 | 0.84 (0.58-1.23) | .377 | 0.66 (0.46-0.94) | | .023 | 0.55 (0.31-0.99) | | .048 |  |
| Chemotherapy |  |  |  |  |  | |  |  | |  |  |
| No | 1.00 |  | 1.00 |  | 1.00 | |  | 1.00 | |  |  |
| Yes | 1.58 (0.50-5.03) | .458 | 0.53 (0.30-0.94) | .030 | 0.51 (0.32-0.82) | | .005 | 0.31 (0.18-0.53) | | <.001 |  |
| Hormonal therapy |  |  |  |  |  | |  |  | |  |  |
| No | 1.00 |  | 1.00 |  | 1.00 | |  | 1.00 | |  |  |
| Yes | 1.20 (0.64-2.23) | .566 | 1.29 (0.80-2.08) | .294 | 1.06 (0.72-1.56) | | .775 | 0.80 (0.48-1.35) | | .406 |  |
| Target therapy |  |  |  |  |  | |  |  | |  |  |
| No | 1.00 |  | 1.00 |  | 1.00 | |  | 1.00 | |  |  |
| Yes | 0.52 (0.18-1.51) | .228 | 0.53 (0.22-1.27) | .153 | 0.39 (0.17-0.86) | | .020 | 0.39 (0.12-1.32) | | .132 |  |

*Abbreviations:* HR = hazard ratio; CI = confidence interval; RNI = regional nodal irradiation; HER2 = human epidermal growth factor receptor 2

Supplementary Table 2. Multivariate analyses of risk factors for outcome events in the high-risk group

| **Variate** | **Locoregional recurrence** | | **Distant metastasis** | | | **Disease-free survival** | | | **Overall survival** | | |
| --- | --- | --- | --- | --- | --- | --- | --- | --- | --- | --- | --- |
|  | **HR (95% CI)** | ***P*** | **HR (95% CI)** | ***P*** | **HR (95% CI)** | | ***P*** | **HR (95% CI)** | | ***P*** |  |
| Year |  |  |  |  |  | |  |  | |  |  |
| 1999-2008 | 1.00 |  | 1.00 |  | 1.00 | |  | 1.00 | |  |  |
| 2009-2014 | 1.63 (0.95-2.81) | .076 | 1.14 (0.74-1.77) | .551 | 1.23 (0.84-1.81) | | .284 | 1.16 (0.68-1.95) | | .586 |  |
| Age (years) |  |  |  |  |  | |  |  | |  |  |
| > 40 | 1.00 |  | 1.00 |  | 1.00 | |  | 1.00 | |  |  |
| ≤ 40 | 1.46 (0.84-2.54) | .182 | 1.41 (0.90-2.23) | .137 | 1.40 (0.93-2.11) | | .102 | 2.03 (1.16-3.54) | | .013 |  |
| Tumor location |  |  |  |  |  | |  |  | |  |  |
| Others | 1.00 |  | 1.00 |  | 1.00 | |  | 1.00 | |  |  |
| Inner quadrant | 1.99 (1.17-3.36) | .010 | 1.86 (1.20-2.89) | .006 | 1.67 (1.13-2.47) | | .010 | 2.06 (1.20-3.51) | | .008 |  |
| T stage |  |  |  |  |  | |  |  | |  |  |
| T1 | 1.00 |  | 1.00 |  | 1.00 | |  | 1.00 | |  |  |
| T2 | 0.84 (0.44-1.60) | .605 | 1.45 (0.81-2.59) | .215 | 1.16 (0.70-1.93) | | .559 | 1.38 (0.68-2.81) | | .376 |  |
| No. of positive nodes |  |  |  |  |  | |  |  | |  |  |
| 1 | 1.00 |  | 1.00 |  | 1.00 | |  | 1.00 | |  |  |
| 2-3 | 1.62 (0.89-2.96) | .117 | 1.94 (1.16-3.25) | .011 | 1.52 (0.98-2.37) | | .062 | 1.88 (1.01-3.50) | | .048 |  |
| Lymphovascular invasion |  |  |  |  |  | |  |  | |  |  |
| No | 1.00 |  | 1.00 |  | 1.00 | |  | 1.00 | |  |  |
| Yes | 1.21 (0.70-2.09) | .497 | 1.51 (0.97-2.36) | .068 | 1.16 (0.77-1.74) | | .468 | 0.82 (0.44-1.53) | | .542 |  |
| Histological grade |  |  |  |  |  | |  |  | |  |  |
| I- II | 1.00 |  | 1.00 |  | 1.00 | |  | 1.00 | |  |  |
| III | 1.57 (0.88-2.81) | .129 | 1.20 (0.76-1.89) | .441 | 0.98 (0.66-1.47) | | .932 | 1.01 (0.57-1.79) | | .971 |  |
| Hormone receptor |  |  |  |  |  | |  |  | |  |  |
| Positive | 1.00 |  | 1.00 |  | 1.00 | |  | 1.00 | |  |  |
| Negative | 1.59 (0.76-3.36) | .219 | 1.71 (0.91-3.22) | .096 | 1.82 (1.04-3.19) | | .036 | 2.06 (0.97-4.39) | | .060 |  |
| HER2 |  |  |  |  |  | |  |  | |  |  |
| Negative | 1.00 |  | 1.00 |  | 1.00 | |  | 1.00 | |  |  |
| Positive | 1.01 (0.58-1.76) | .965 | 1.28 (0.82-2.01) | .280 | 1.09 (0.73-1.63) | | .681 | 1.30 (0.75-2.26) | | .352 |  |
| Surgery type |  |  |  |  |  | |  |  | |  |  |
| Breast-conserving | 1.00 |  | 1.00 |  | 1.00 | |  | 1.00 | |  |  |
| Mastectomy | 1.74 (0.67-4.53) | .253 | 1.03 (0.50-2.10) | .938 | 1.12 (0.59-2.14) | | .722 | 0.98 (0.43-2.21) | | .955 |  |
| RNI |  |  |  |  |  | |  |  | |  |  |
| No | 1.00 |  | 1.00 |  | 1.00 | |  | 1.00 | |  |  |
| Yes | 0.56 (0.31-1.00) | .050 | 0.75 (0.47-1.18) | .217 | 0.73 (0.48-1.10) | | .133 | 0.78 (0.44-1.38) | | .390 |  |
| Chemotherapy |  |  |  |  |  | |  |  | |  |  |
| No | 1.00 |  | 1.00 |  | 1.00 | |  | 1.00 | |  |  |
| Yes | 1.51 (0.36-6.27) | .571 | 2.06 (0.50-8.49) | .317 | 1.24 (0.45-3.40) | | .680 | 0.74 (0.22-2.45) | | .626 |  |
| Hormonal therapy |  |  |  |  |  | |  |  | |  |  |
| No | 1.00 |  | 1.00 |  | 1.00 | |  | 1.00 | |  |  |
| Yes | 0.91 (0.46-1.82) | .799 | 0.74 (0.41-1.34) | .322 | 0.86 (0.51-1.44) | | .573 | 0.57 (0.28-1.14) | | .112 |  |
| Target therapy |  |  |  |  |  | |  |  | |  |  |
| No | 1.00 |  | 1.00 |  | 1.00 | |  | 1.00 | |  |  |
| Yes | 0.41 (0.17-1.02) | .055 | 0.40 (0.18-0.85) | .018 | 0.42 (0.22-0.82) | | .011 | 0.23 (0.07-0.77) | | .017 |  |

*Abbreviations:* HR = hazard ratio; CI = confidence interval; RNI = regional nodal irradiation; HER2 = human epidermal growth factor receptor 2

Supplementary Table 3. Multivariate analyses of risk factors for outcome events in the low-risk group

| **Variate** | **Locoregional recurrence** | | **Distant metastasis** | | | **Disease-free survival** | | | **Overall survival** | | |
| --- | --- | --- | --- | --- | --- | --- | --- | --- | --- | --- | --- |
|  | **HR (95% CI)** | ***P*** | **HR (95% CI)** | ***P*** | **HR (95% CI)** | | ***P*** | **HR (95% CI)** | | ***P*** |  |
| Year |  |  |  |  |  | |  |  | |  |  |
| 1999-2008 | 1.00 |  | 1.00 |  | 1.00 | |  | 1.00 | |  |  |
| 2009-2014 | 1.15 (0.51-2.61) | .734 | 0.94 (0.58-1.54) | .818 | 0.83 (0.54-1.29) | | .410 | 0.74 (0.39-1.41) | | .364 |  |
| Age (years) |  |  |  |  |  | |  |  | |  |  |
| > 40 | 1.00 |  | 1.00 |  | 1.00 | |  | 1.00 | |  |  |
| ≤ 40 | 0 (0-5.72E+303) | .975 | 1.36 (0.45-4.06) | .585 | 1.24 (0.42-3.66) | | .694 | 1.60 (0.34-7.60) | | .556 |  |
| Tumor location |  |  |  |  |  | |  |  | |  |  |
| Others | 1.00 |  | 1.00 |  | 1.00 | |  | 1.00 | |  |  |
| Inner quadrant | 4.17 (1.17-14.85) | .028 | 2.08 (0.97-4.46) | .061 | 2.26 (1.14-4.50) | | .020 | 3.22 (1.15-9.01) | | .026 |  |
| T stage |  |  |  |  |  | |  |  | |  |  |
| T1 | 1.00 |  | 1.00 |  | 1.00 | |  | 1.00 | |  |  |
| T2 | 4.71 (1.46-15.25) | .010 | 2.27 (1.17-4.37) | .015 | 2.40 (1.32-4.35) | | .004 | 3.40 (1.40-8.24) | | .007 |  |
| No. of positive nodes |  |  |  |  |  | |  |  | |  |  |
| 1 | 1.00 |  | 1.00 |  | 1.00 | |  | 1.00 | |  |  |
| 2-3 | 1.40 (0.35-5.61) | .635 | 1.13 (0.56-2.31) | .728 | 1.38 (0.74-2.57) | | .311 | 1.55 (0.60-4.04) | | .368 |  |
| Lymphovascular invasion |  |  |  |  |  | |  |  | |  |  |
| No | 1.00 |  | 1.00 |  | 1.00 | |  | 1.00 | |  |  |
| Yes | 2.96 (0.33-26.67) | .333 | 2.38 (0.69-8.18) | .169 | 1.96 (0.58-6.65) | | .277 | 3.32 (0.70-15.8) | | .132 |  |
| Histological grade |  |  |  |  |  | |  |  | |  |  |
| I- II | 1.00 |  | 1.00 |  | 1.00 | |  | 1.00 | |  |  |
| III | 1.15 (0.13-10.38) | .898 | 1.44 (0.53-3.93) | .475 | 1.29 (0.48-3.45) | | .613 | 3.17 (0.94-10.75) | | .064 |  |
| Hormone receptor |  |  |  |  |  | |  |  | |  |  |
| Positive | 1.00 |  | 1.00 |  | 1.00 | |  | 1.00 | |  |  |
| Negative | 7.69 (0.41-143.04) | .171 | 2.55 (0.79-8.23) | .118 | 2.98 (1.08-8.27) | | .035 | 4.54 (1.33-15.53) | | .016 |  |
| HER2 |  |  |  |  |  | |  |  | |  |  |
| Negative | 1.00 |  | 1.00 |  | 1.00 | |  | 1.00 | |  |  |
| Positive | 7.03 (1.28-38.60) | .025 | 0.78 (0.10-5.84) | .806 | 1.34 (0.31-5.74) | | .693 | 0 (0-7.77E+214) | | .972 |  |
| Surgery type |  |  |  |  |  | |  |  | |  |  |
| Breast-conserving | 1.00 |  | 1.00 |  | 1.00 | |  | 1.00 | |  |  |
| Mastectomy | 1.27 (0.37-4.43) |  | 0.88 (0.46-1.68) | .703 | 1.06 (0.56-1.99) | | .859 | 0.87 (0.35-2.12) | | .753 |  |
| RNI |  |  |  |  |  | |  |  | |  |  |
| No | 1.00 |  | 1.00 |  | 1.00 | |  | 1.00 | |  |  |
| Yes | 0.29 (0.04-2.15) | .226 | 0.66 (0.28-1.55) | .342 | 0.63 (0.29-1.38) | | .249 | 0.78 (0.27-2.25) | | .651 |  |
| Chemotherapy |  |  |  |  |  | |  |  | |  |  |
| No | 1.00 |  | 1.00 |  | 1.00 | |  | 1.00 | |  |  |
| Yes | 2.26 (0.30-16.77) | .426 | 1.51 (0.55-4.17) | .427 | 0.85 (0.42-1.70) | | .643 | 0.38 (0.17-0.84) | | .017 |  |
| Hormonal therapy |  |  |  |  |  | |  |  | |  |  |
| No | 1.00 |  | 1.00 |  | 1.00 | |  | 1.00 | |  |  |
| Yes | 2.25 (0.30-16.73) | .429 | 0.56 (0.28-1.14) | .112 | 0.54 (0.29-1.01) | | .053 | 0.30 (0.14-0.64) | | .002 |  |
| Target therapy |  |  |  |  |  | |  |  | |  |  |
| No | 1.00 |  | 1.00 |  | 1.00 | |  | 1.00 | |  |  |
| Yes | 1.01 (0.09-11.48) | .992 | 4.78 (0.43-53.20) | .203 | 2.70 (0.38-19.3) | | .324 | 0 (0-1.15E+243) | | .973 |  |

*Abbreviations:* HR = hazard ratio; CI = confidence interval; RNI = regional nodal irradiation; HER2 = human epidermal growth factor receptor 2
